# Validation of Automated Bacterial Suspension Preparation by Colibri^®^ and Plate Streaking by WASP^®^ for Antibiotic Disk Diffusion Susceptibility Testing

**DOI:** 10.3390/antibiotics14121178

**Published:** 2025-11-21

**Authors:** Robin Vanstokstraeten, Eveline Van Honacker, Kristof Emmerechts, Zan Janssen, Charlotte Michel, Goran Van Der Kelen, Kristof Vandoorslaer, Deborah De Geyter, Bram Vanmechelen

**Affiliations:** 1 Department of Microbiology and Infection Control, Universitair Ziekenhuis Brussel (UZ Brussel), 1090 Brussels, Belgium; 2 Vitality Research Group, Vrije Universiteit Brussel (VUB), 1090 Brussels, Belgium; 3 Faculty of Medicine and Pharmacy, Vrije Universiteit Brussel (VUB), 1090 Brussels, Belgium

**Keywords:** total lab automation, WASPLab, Colibri, WASP, Radian, disk diffusion, antibiotic susceptibility testing

## Abstract

Background/objectives: One of the most crucial responsibilities of clinical microbiology laboratories involves conducting precise and fast antimicrobial susceptibility testing (AST) on bacterial isolates, necessary to guide antibiotic therapy. Standardized disk diffusion, a manual AST method, consumes a significant amount of time and is error-prone. Total laboratory automation in microbiology should enable a lower workload, high traceability, and standardization in AST. Therefore, we examined the concordance at the categorical level between the manual reference method and a new automated approach for bacterial suspension preparation and plate streaking in AST. Methods: In this study, we validated the automated bacterial suspension preparation by Colibri^®^ and plate streaking by WASP^®^ for antibiotic disk diffusion susceptibility testing. Two hundred and one non-duplicate bacterial strains, derived from a variety of different bacterial species, encompassing key known resistance mechanisms and comprising both Gram-positive (N = 78) and Gram-negative (N = 123) strains, were tested. Both the manual (reference) and the automated (Colibri^®^ with WASP^®^) method for AST preparation and plate streaking used the Radian^®^ in-line carousel and expert system for antibiotic susceptibility interpretation. European Committee on Antimicrobial Susceptibility Testing clinical breakpoints (version 13.1) were used to interpret susceptibility results. Results: The overall categorical agreement between the two compared methods was 96.3% (2186/2269). We identified 2.7% (62/2269) minor errors, 1.6% (17/1047) major errors, and 0.4% (4/1121) very major errors. However, it is noteworthy that after retesting the discrepant results, the major errors were reduced to 0.4% and the very major errors were reduced to 0%. Conclusions: The combination of Colibri^®^ and WASP^®^ appears to be a compelling automated tool for the automated preparation of bacterial suspensions and plate streaking in AST, with an accuracy that is equal to the reference method. Furthermore, it enables the optimization of hands-on time and standardization of (pre-) analytical procedures.

## 1. Introduction

One of the most crucial responsibilities of clinical microbiology laboratories involves conducting precise and fast antimicrobial susceptibility testing (AST) on bacterial isolates, necessary to guide antibiotic therapy. Standardized disk diffusion, a manual method that is frequently used for AST, consumes a significant amount of time and is error-prone. Moreover, a substantial proportion of published AST reports either do not adjust to the recommended 0.5 McFarland standard or fail to describe how inoculum density was standardized, highlighting the need for rigorous standardization in both manual and automated workflows [1,2].

In the past decade, clinical microbiology laboratories have witnessed enhanced productivity, traceability, standardization, and quality through the implementation of laboratory automation [3]. A notable example of this advancement is the introduction of matrix-assisted laser desorption/ionization time of flight mass spectrometry (MALDI-TOF MS), which stands out as one of the most significant breakthroughs in medical microbiology [4]. Moreover, automated clinical specimen processors, picking systems, disk dispensing, and AST-halo interpretation are revolutionizing the field rapidly.

Traditionally, clinical microbiology has been a labor-intensive discipline, requiring manual handling of specimens, cultures, and assays. However, this discipline is rapidly evolving, as total laboratory automation (TLA) is progressively making its entry into routine clinical practices. TLA involves the integration of various automated systems and robotics to streamline and optimize the entire laboratory process, from specimen handling to result reporting. Two great examples of systems providing TLA in clinical microbiology are WASPLab^®^ (Copan Diagnostics, Brescia, Italy) and BD Kiestra^®^ (Drachten, The Netherlands) [3]. Built upon the foundational techniques utilized in clinical microbiology laboratories, these systems involve the automated processes of specimen streaking, incubation, sample transport, and imaging, particularly focusing on the cultivation of bacterial colonies on agar plates. Yet, the implementation of automation in microbiology is not as conspicuous as it is in hematology or chemistry. Indeed, the diverse range of clinical specimens and container types, the intricacy of analytical procedures, and the diversity of diagnostic methods presented significant challenges to enacting automation in the clinical microbiology laboratory [5].

Copan, a commercial company, is pushing the boundaries of clinical microbiology TLA by incorporating various modules that seamlessly integrate with WASPLab^®^, helping clinicians make rapid therapeutic decisions [6]. Take Colibri^®^, for instance, a device designed to automatize colony picking, and preparation of targets for the identification of bacteria and yeasts through MALDI-TOF technology, and bacterial suspensions for AST [7,8]. The Colibri coupled with the Radian in-Line Carousel^®^ and the Radian Expert System^®^ for antibiotic disk dispensing and halo interpretation, provides a fully automated solution for antimicrobial disk diffusion susceptibility testing [9,10].

In this study, we validated the automated bacterial suspension preparation using Colibri^®^ and plate streaking by WASP^®^, the automated specimen processor within WASPLab^®^, for antibiotic disk diffusion susceptibility testing. To verify the performance of this novel automated workflow for AST, it is crucial to analyze a diverse set of clinical strains resistant to different antibiotic agents. Hence, we validated this modus operandi by testing a plethora of different clinically relevant bacterial species, encompassing key known resistance mechanisms and comprising both Gram-positive and Gram-negative strains.

## 2. Results

Among the 201 strains tested, the categorical agreement (CA) between the two methods was initially 96.3% (2186/2269). Minor errors accounted for 2.7% (62/2269), major errors for 1.6% (17/1047), and very major errors for 0.4% (4/1121) (Supplementary Data). After retesting all major and very major results, the CA increased to 97.0%, resulting in an acceptable overall CA (Table 1). Minor errors were not retested.

All four very major errors were found in Gram-positive strains: one for cefoxitin in a *Staphylococcus epidermidis* strain (reference: Q23/023), one for moxifloxacin in a *Staphylococcus aureus* strain (Q23/064) and both linezolid and vancomycin in the same *Enterococcus faecalis* strain (REF-814) (Supplementary Data). Upon repeat testing of these discrepant cases, all four results were found to be concordant, thereby eliminating the initial very major errors (Table 2).

Similarly, of the 17 initial major errors, repeat testing confirmed only four as true major errors, with the remainder demonstrating concordant results. Three of these remaining major errors were found in Gram-negative bacteria and one in a Gram-positive bacterium: one for moxifloxacin in an *Enterobacter cloacae* strain (reference: Q23/013), one for gentamicin in a *Klebsiella aerogenes* strain (REF-793), one for piperacillin/tazobactam in a *Klebsiella oxytoca* strain (REF-716) and one for moxifloxacin in a *Staphylococcus aureus* strain (Q21/003) (Supplementary Data) (Table 2).

**Figure 1:**
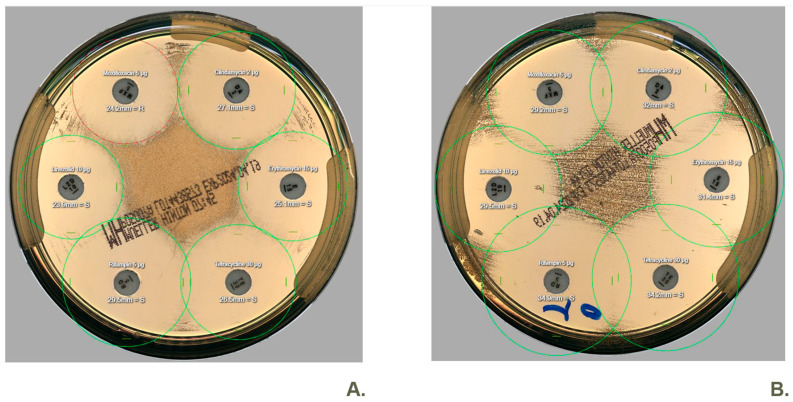
Two images depicting disk diffusion of a clinical *Staphylococcus aureus* isolate on Mueller-Hinton agar were captured using WASPLab^®^. Both images, labeled (**A**,**B**), utilized the Radian in-Line Carousel^®^ and the Radian Expert System^®^ for antibiotic disk dispensing and halo interpretation. Plate streaking and suspension preparation were conducted by WASP^®^ in image (**A**), whereas they were performed manually in image (**B**). In this case, we observed a discrepancy (ME) where moxifloxacin is classified as resistant (red halo) according to the new method but susceptible (green halo) according to the standard/reference method.

After retesting, the outcomes obtained with the new automated Colibri^®^ methodology tend to remain consistent in 12 of the 19 retests (Table 2). Overall, more confluent growth was observed when using the novel method (Figure 1). It is plausible that some of the discrepancies may be attributable to this factor.

## 3. Discussion

Clinical microbiology is rapidly evolving, with manual and often labor-intensive methods being replaced by TLA, aiming for a lower workload, higher traceability and better standardization. In this study, we validated the automated bacterial suspension preparation by Colibri^®^ and plate streaking on a large cohort of non-duplicate bacterial strains (N = 201) with a plethora of different resistance mechanisms by WASP^®^ for antibiotic disk diffusion susceptibility testing.

Although automation in medical microbiology remains less prevalent compared to clinical chemistry and hematology, TLA is steadily gaining traction in routine microbiological diagnostics. Several integrated systems are already available, incorporating automated inoculators, labeling devices, incubators, imaging systems, and transport modules, all operating with a unified workflow [3]. However, one component that has yet to be fully validated, and consequently is not yet routinely incorporated into TLA, is a system for fully automated AST. Copan has developed and introduced such an automated AST platform within its TLA framework, known as WASPLab^®^, representing significant advancement toward fully automated microbiology workflows [6]. This AST platform is called “Radian”, and consists of the Radian^®^ in-line carousel (hardware) and the Radian^®^ expert system (software) [9,10].

Prior to this validation, some other studies already mentioned the Radian^®^ in-line Carousel and expert system, and Colibri^®^. In 2021, Cherkaoui et al. assessed the agreement at the categorical level between the Vitek 2 system and Colibri coupled to the Radian^®^ under real routine laboratory conditions. They included 675 non-duplicate clinical strains and described an impressive 99.3%, 98.6%, 99.4% and 99.4% (CA) between the two methods for *Enterobacterales*, *P. aeruginosa*, *Staphylococcus* spp. and *Enterococcus* spp., respectively [9].

Shortly thereafter, in 2022, Herroelen et al. evaluated the automation of the EUCAST rapid AST (RAST) method for positive blood culture bottles using WASPLab^®^, inclusive Colibri^®^ and Radian^®^ in Line Carousel. This study confirms that integrating EUCAST RAST with the WASPLab^®^ system, including Colibri^®^ and the Radian^®^ carousel, provides accurate and reproducible rapid AST results directly from positive blood cultures, significantly reducing turnaround time and supporting timely clinical decision-making [11]. Recently, in 2024, Callebaut et al. assessed the CA between standardized disk diffusion (reference method) and Radian^®^ using EUCAST 2021 breakpoints. They observed high CA of 95.3%, 96.3%, 93.8%, 97.3% and 98.0% for *Enterobacterales*, *Enterococcus* spp., *P. aeruginosa*, *Staphylococcus* spp. and *Streptococcus* spp., respectively [10].

When validating the automated bacterial suspension preparation by Colibri^®^ and plate streaking by WASP^®^ for AST, we obtained very similar results like in the above-mentioned studies. Without retesting discrepant results, we obtained an overall CA of 96.3% (2186/2269). Only 0.4% (4/1121) very major errors were found, which were found to be concordant after retesting them. Similarly, of the 17 initial major errors, repeat testing confirmed only four as true major errors, with the remainder demonstrating concordant results. Retesting of these discrepancies was performed using freshly cultured isolates. Upon review, no mixed or morphologically distinct colonies were observed. Nevertheless, potential heteroresistance remains an important consideration when interpreting discordant results [12]. Additionally, some discrepancies may be related to inoculum effects, which are known to influence susceptibility results for certain antibiotic classes such as fluoroquinolones, carbapenems, and glycopeptides [13]. Finally, some discrepancies may be attributable to the fact that WASP^®^ is generally more homogeneous and standardized compared to manual streaking (Figure 1) [14]. Consequently, inhibition zones are typically more clearly defined. Furthermore, the manual delineation of disk diffusion zones may also contribute to these differences. Although the software typically performs automatic zone measurements, occasional manual adjustments by laboratory personnel remain necessary in some cases. In this study, no issues were observed with specific species or colony morphologies, including mucoid, swarming, adherent, or small/friable colonies, indicating that the automated workflow handled all the phenotypes successfully.

It should also be acknowledged that certain discrepancies fall within the range of analytical or technical uncertainty (ATU) [15]. Additionally, this study did not account for heteroresistant strains in cases of discordant results (e.g., colonies visible within the inhibition zone of disk diffusion), which are a recognized source of errors, particularly in species such as *Pseudomonas aeruginosa* [16].

Another limitation of this study is the absence of broth microdilution as the reference standard. Disk diffusion was used for comparison, but is not considered the definitive gold standard model. This limitation is fully recognized, as broth microdilution represents the most standardized and quantitative reference in accordance with ISO guidelines [17]. Disk diffusion was chosen as the comparator because it reflects the current routine workflow in the authors’ laboratory. Therefore, the resulting major and very major error rates should be interpreted as method-to-method agreement, rather than ISO-standard accuracy estimates. Future work should therefore aim to incorporate broth microdilution to enhance the robustness and comparability of automated disk diffusion validation [18].

Finally, it should be recognized that this study does not provide quantitative data on the difference in hands-on time and total turnaround time (TAT) between the automated and manual workflows. While automation clearly reduces the need for manual tasks—such as streaking agar plates, placing disks, and measuring inhibition zones—the exact time savings are difficult to determine. This represents a limitation of this study, and future work could aim to systematically measure and compare hands-on time and TAT across different workflows. However, it is important to note that a faster TAT is not necessarily the primary goal of automation in microbiology; rather, the main objectives are to reduce hands-on time, increase standardization, and improve traceability within the workflow.

## 4. Materials and Methods

### 4.1. Bacterial Strains

This study was performed in the clinical microbiology laboratory of the University Hospital Brussels, a Belgian tertiary care center with more than 700 beds. A total of 201 non-duplicate bacterial strains were included encompassing 78 Gram-positive and 123 Gram-negative strains, consisting of both reference strains (ATCC and NTCT strains) and strains isolated from clinical samples (blood cultures) stored at −80 °C with 15% glycerol (Supplementary Data).

The Gram-positive strains included *Enterococcus faecalis* (N = 5), *Enterococcus faecium* (N = 15), *Enterococcus gallinarum* (N = 1), *Staphylococcus aureus* (N = 24), *Staphylococcus epidermidis* (N = 10), *Staphylococcus haemolyticus* (N = 4), *Staphylococcus hominis* (N = 2), *Staphylococcus lugdunensis* (N = 4), *Staphylococcus warneri* (N = 1), *Streptococcus agalactiae* (N = 8), and *Streptococcus pyogenes* (N = 4) (Supplementary Data). The Gram-negative strains included *Acinetobacter baumannii* complex (N = 3), *Acinetobacter species* (N = 1), *Citrobacter freundii complex* (N = 3), *Citrobacter koseri* (N = 1), *Enterobacter cloacae complex* (N = 7), *Escherichia coli* (N = 34), *Klebsiella aerogenes* (N = 3), *Klebsiella oxytoca* (N = 3), *Klebsiella oxytoca/Raoultella* species (N = 2), *Klebsiella pneumoniae* (N = 32), *Morganella morganii* (N = 3), *Proteus mirabilis* (N = 1), *Proteus vulgaris* (N = 1), *Pseudomonas aeruginosa* (N = 24), *Pseudomonas putida* (N = 1), *Salmonella* species (N = 1) and *Serratia marcescens complex* (N = 3) (Supplementary Data).

The following confirmed resistance mechanisms and phenotypes were included: extended-spectrum beta-lactamase (ESBL) (N = 25), imipenemase (IMP) (N = 1), Verona-integron metallo-beta-lactamase (VIM) (N = 9), methicillin-resistant *Staphylococcus aureus* (MRSA) (N = 15), New Delhi metallo-beta-lactamase (NDM) (N = 11), oxacillinase-48 (OXA-48) (N = 15), *Klebsiella pneumoniae* carbapenemase (KPC), *vanA* gene (N = 6), *vanB* gene (N = 4), *vanC1* gene (N = 1) (Supplementary Data).

### 4.2. Identification of Species

MALDI-TOF MS was performed prior to strain preservation at −80 °C, using the Biotyper^®^ (Bruker Daltonics, Bremen, Germany). A single colony was placed on a polished steel MALDI MSP 96 target plate (Bruker Daltonics, Bremen, Germany). The colony was then treated with 1 µL of formic acid, allowed to air-dry at room temperature, and subsequently covered with 1 µL of a matrix solution containing α-cyano-4-hydroxycinnamic acid (10 mg/mL) in a solvent mixture of 50% acetonitrile, 47.5% water, and 2.5% trifluoroacetic acid (Bruker Daltonics, Bremen, Germany). After drying at room temperature, the samples were analyzed using the MALDI Biotyper^®^ Sirius system (Bruker Daltonics, Bremen, Germany), which is equipped with the Smartbeam MBT version GFLIC-2 laser in positive-ion mode. Spectra were acquired using the FlexControl software version 3.4 build 207.20 from the manufacturer. Isolates that achieved an identification score above 2.0 with a single unique hit were considered accurately identified according to the MALDI MBT compass version 4.1 build 100.

### 4.3. Identification of Resistance Mechanisms and Phenotypes

The detection of OXA-48, KPC, NDM, VIM, and IMP enzymes was carried out prior to strain preservation at −80 °C using the RESIST-5 O.K.N.V.I. immunochromatographic lateral flow assay (Coris BioConcept, Gembloux, Belgium). The EUCAST disk diffusion method for phenotypic detection of ESBL was conducted on Mueller-Hinton agar (I2A, Montpellier, France) using ceftazidime, ceftriaxone, cefepime and corresponding combinations with clavulanic acid. The Mueller-Hinton agar plates were incubated at 37 °C for 24 h.

The interpretation of disk diffusion zone diameters was based on the European Committee on Antimicrobial Susceptibility Testing (EUCAST) guidelines [19]. Initial screening of MRSA was performed using BBL CHROMagar MRSA II (Becton Dickson, Drachten, The Netherlands), and confirmation of methicillin resistance was achieved via disk-diffusion testing, with resistance to cefoxitin indicating MRSA [20]. Vancomycin-resistant genotypes *(vanA*, *vanB*, *vanC*) were detected by using an in-house multiplex PCR.

### 4.4. Antimicrobial Susceptibility Testing

Thawed bacterial strains were manually inoculated onto a sheep blood agar plate supplemented with X and V factors (referred to as a HEM plate, prepared in-house), in accordance with the standard operating procedures of the laboratory. The plates were incubated aerobically at 35 °C for 24 h until sufficient growth was obtained to prepare a 0.5 McFarland suspension in a 0.9% saline, as required for disk diffusion testing in both manual and automated workflows, according to CLSI and EUCAST guidelines [21,22]. This suspension should ideally be used for inoculation within 15 min, and in all cases within 60 min. In our routine manual practice, an inoculum turbidity between 0.4 and 0.6 McFarland is generally considered acceptable; for the automated workflow using Colibri^®^, the density or McFarland value of the suspension is not displayed. However, all tubes considered valid contain a microbial concentration within the range validated by the manufacturer.

**Figure 2:**
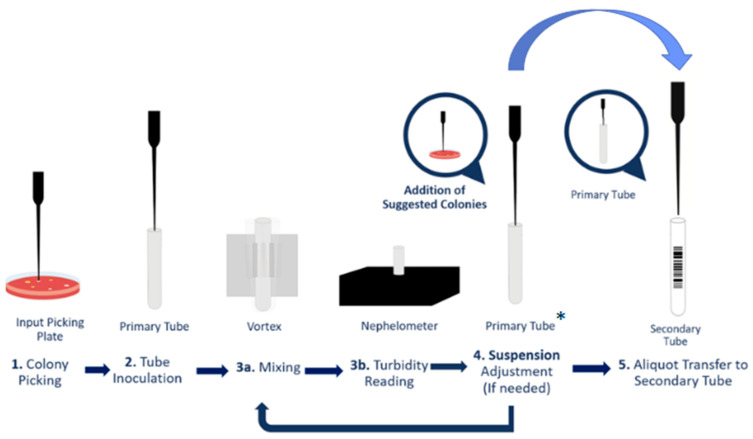
Schematic summary of the Colibri AST-workflow. * The concentration of the primary tube is variable, it is calculated based on the concentration of the secondary tube, in order to reach the concentration of the secondary tube required by the downstream instrument. The concentration of the secondary tube corresponds to a 1:3 dilution of the 0.5 McFarland standard, which ensures the same bacterial concentration on the AST plate as obtained with the manual method, where a 0.5 McFarland suspension is streaked using a cotton swab.

In the automated disk diffusion method using WASP^®^, the required concentration corresponds to a 1:3 dilution of the 0.5 McFarland standard. This dilution ensures the same bacterial concentration on the AST plate as obtained with the manual method, where a 0.5 McFarland suspension is streaked using a cotton swab. The Colibrí^®^ system can pick isolated colonies directly from the HEM plate and prepare a heavy suspension in the primary tube. The concentration of this suspension is then checked by an on-board nephelometer, and the heavy suspension is automatically diluted in a secondary tube to the concentration required by the downstream AST preparator (Figure 2).

Both the automated and manual methods were initiated from the same frozen bacterial stock, ensuring that identical strains were used across all tests. During colony selection, whether performed manually or by the Colibri^®^ system, colonies were visually inspected for morphological uniformity. In cases where mixed or morphologically distinct colonies were observed, the corresponding culture plate, suggesting potential strain heterogeneity, was excluded from the validation. The automated AST was carried out using Copan’s Colibri^®^ and WASP^®^, both modules of the TLA WASPLab^®^ system (Copan, Brescia, Italy). Colibri^®^ was employed to prepare the microbial suspension for AST following pre-selection of colonies from the HEM plates using the WASPLab^®^ Webapp. The Colibri^®^ pipetting system then directly selects the prechosen colonies and transfers a standardized amount of colony material for 0.5 McFarland suspension preparation. A one-third dilution of the suspension was prepared in phosphate-buffered saline and subsequently used as the inoculum. Subsequently, the WASP^®^ was used to streak 60 µL of this inoculum on a round 90 mm Mueller-Hinton agar plate (Thermo Fisher Scientific, Waltham, MA, USA). Mueller-Hinton agar was also streaked manually with a 0.5 McFarland suspension prepared by hand, following EUCAST guidelines for disk diffusion as the reference method [22,23].

Both the manual (reference) and automated (Colibri^®^ with WASP^®^) methods for AST preparation and plate streaking utilized the Radian^®^ in-line carousel and expert system for interpreting antibiotic susceptibility. Colibri^®^ and WASP^®^ were used precisely according to the manufacturer’s instructions. The inoculated agar plates were incubated aerobically for 18 h at 35 °C in the WASPLab^®^. After exactly 18 h, high-resolution images of the disk diffusion were captured under various lighting conditions in accordance with the manufacturer’s guidelines.

The in-line carousel can hold up to 50 different antibiotic cartridges and supports up to eight antibiotic disk deposition protocols [24]. The expert system is a software integrated with the WASPLab^®^ Webapp, enabling automatic measurement of inhibition zone diameters and interpretation according to EUCAST or Clinical and Laboratory Standards Institute (CLSI) guidelines. In this study, we used eight different antibiotic disk deposition protocols, depending on the class of bacteria (e.g., Gram-negative bacilli or Gram-positive cocci). A total of 29 distinct types of antibiotic disks were utilized across the eight different antibiotic disk deposition protocols (Table 1). Using the Radian^®^ In-Line Carousel, six antibiotic disks were dispensed per Mueller-Hinton agar plate. The disk configuration per protocol is consultable in the Supplementary Data.

**Figure 3:**
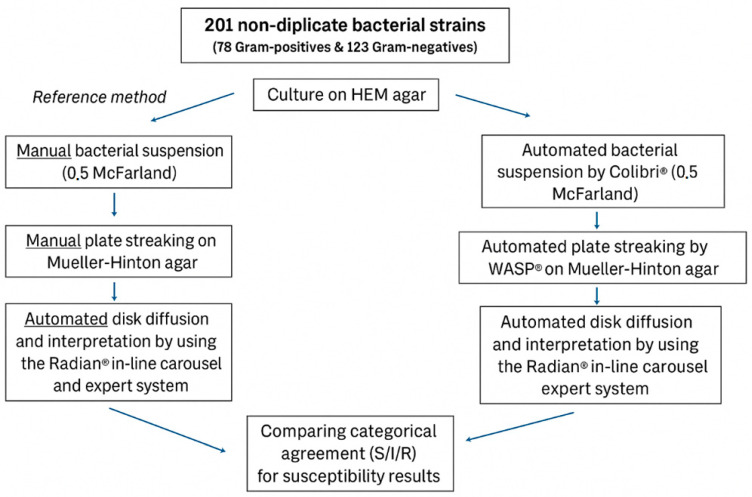
A simplified overview of the study protocol. AST was performed on all 201 non-duplicate isolates twice: once with the reference method (left column) and once with the fully automated new method (right column). HEM agar is an in-house made agar based on sheep blood and growth factors X and V. The categorical outcomes for susceptibility were compared to determine the categorical agreement. S: susceptible, I: susceptible, increased exposure, R: resistant.

All antibiotic cartridges (i2a, Montpellier, France) were stored at 4 °C in accordance with the manufacturer’s instructions and were loaded into the Radian carousel only when conducting the AST. In this study, EUCAST clinical breakpoints (version 13.1) were used by the Radian^®^ expert system to interpret susceptibility results [19]. All the final AST results were validated by an experienced lab technologist. Halo adjustments were maintained to a minimum: the technologist needed to make corrections in less than 1% of the validated results. A simplified overview of AST protocol can be found in Figure 3. 

### 4.5. Quality Control

Quality control was performed throughout the study by testing American Type Culture Collection (ATCC) and National Collection of Type Cultures (NCTC) quality control organisms across all testing days. A detailed list of the strains used, along with their inclusion dates, is provided in the Supplementary Data.

### 4.6. Statistical Analysis and Discordant Results

The categorization of agreement, including the identification of minor errors (mEs), major errors (MEs), and very major errors (VMEs), was performed according to the ISO 20776-2 standard [17]. The potential categorical outcomes of the AST were defined as susceptible (S), susceptible with increased exposure (I), and resistant (R). In this study, AST performed using a manually prepared bacterial suspension and manual plate streaking served as the reference method.

VMEs were defined as cases where the new method classified an isolate as susceptible, while the reference method classified it as resistant. This represents the most critical error type, as it could result in the selection of an ineffective antibiotic, potentially leading to treatment failure. ME occurred when the new method classified an isolate as resistant, but the reference method classified it as susceptible, posing a significant risk of inappropriate treatment decisions.

Minor errors were identified when one method categorized an isolate as intermediate, while the other method categorized it as either susceptible or resistant. Although mEs are less critical than MEs and VMEs, they still indicate a discrepancy between the two methods that could influence clinical decision-making. According to the internationally accepted performance standards for AST method evaluations, the following criteria are generally applied as acceptable limits: CA ≥ 90%, VME ≤ 1.5%, ME < 3% and mE < 10% [25,26].

## 5. Conclusions

As laboratories embrace automation, they embark on a journey not only towards addressing the challenges of infectious disease diagnostics but also redefining the standards of precision, speed, and resource utilization in the ever-evolving landscape of clinical microbiology. Through this validation, we contribute to the fast evolving landscape of TLA in medical microbiology.

The integration of Colibri^®^ and WASP^®^ represents a robust and reliable automated solution for the preparation of bacterial suspensions and plate streaking in AST, demonstrating accuracy comparable to that of the reference method. Moreover, this approach facilitates the optimization of hands-on time and promotes the standardization of pre-analytical workflows, thereby supporting enhanced efficiency and reproducibility in clinical microbiology and diagnostics. Future multicenter validation will be important to confirm reproducibility across different laboratory settings and to support broader implementation of automated disk diffusion.

## Figures and Tables

**Table 1:** Summary of the S/I/R concordance of the 2269 disk diffusion tests, encompassing 29 different antibiotic disks and 201 unique bacterial isolates, after re-evaluating all the very major and major errors. Antibiotic concentrations are expressed in µg. Abbreviations: CA, categorical agreement; mE, minor error; ME, major error; VME, very major error; NA, not applicable (e.g., amoxicillin/clavulanic acid 30 for *Pseudomonas aeruginosa*).

	S/I/R Agreement					
	CA	mE	ME	VME	NA	Total
**Amikacin 30**	123	0	0	0	0	123
**Amoxicillin/clavulanic acid 30**	93	0	0	0	207	93
**Ampicillin 2**	18	0	0	0	14	18
**Ampicillin 10**	94	0	0	0	2	94
**Aztreonam 30**	110	9	0	0	0	119
**Cefadroxil 30**	94	0	0	0	2	94
**Cefepime 30**	101	18	0	0	0	119
**Cefoxitin 30**	43	0	0	0	2	43
**Ceftazidime 10**	111	8	0	0	0	119
**Ceftriaxone 30**	91	3	0	0	2	94
**Cefuroxime 30**	73	0	0	0	17	73
**Ciprofloxacin 5**	173	10	0	0	1	183
**Clindamycin 2**	54	0	0	0	2	54
**Erythromycin 15**	54	0	0	0	2	54
**Fosfomycin 200**	34	0	0	0	30	34
**Gentamicin 10**	140	0	1	0	4	141
**Gentamicin 30**	0	0	0	0	16	0
**Linezolid 10**	72	0	0	0	0	72
**Meropenem 10**	109	14	0	0	0	123
**Moxifloxacin 5**	145	0	2	0	4	147
**Nitrofurantoin 100**	43	0	0	0	46	43
**Oxacillin 1**	0	0	0	0	16	0
**Penicillin G 1**	38	0	0	0	3	38
**Piperacillin/tazobactam 36**	30	1	1	0	0	32
**Rifampicin 5**	54	0	0	0	2	54
**Temocillin 30**	72	0	0	0	18	72
**Tetracycline 30**	54	0	0	0	2	54
**Trimethoprim/sulfamethoxazole 25**	150	0	0	0	4	150
**Vancomycin 5**	29	0	0	0	14	29
**Total**	2202	63	4	0	410	2269
**%**	97.0%	2.8%	0.4%	0.0%	/	/

**Table 2:** Listing of all the initial (before retesting) very major errors (VME) and major errors (ME) with an overview of the characteristics. The shaded rows are errors that persisted after retesting, and are considered ‘true’ ME. Strain references can be found in the supplementary data. *Amikacin showed a concordant result after retesting (Colibri R, manual R).

Strain Reference	Species	Type of Error	Discrepant Antibiotic(s)	Results Before Rerun	Results After Rerun
**Q23/023**	*S. epidermidis*	VME	cefoxitin	Colibri S, manual R	Colibri S, manual S
**Q23/064**	*S. aureus*	VME	moxifloxacin	Colibri S, manual R	Colibri S, manual S
**REF-814**	*E. faecalis*	VME	linezolid, vancomycin	Colibri S, manual R	Colibri S, manual S
**Q21/003**	*S. aureus*	ME	moxifloxacin	Colibri R, manual S	Colibri R, manual S
**Q22/035**	*E. faecium*	ME	linezolid	Colibri R, manual S	Colibri S, manual S
**REF-739**	*S. pyogenes*	ME	penicillin	Colibri R, manual S	Colibri S, manual S
**Q23/013**	*E. cloacae*	ME	moxifloxacin	Colibri R, manual S	Colibri R, manual S
**REF-727**	*E. cloacae*	ME	moxifloxacin	Colibri R, manual S	Colibri R, manual R
**REF-793**	*K. aerogenes*	ME	amikacin*, gentamicin	Colibri R, manual S	Colibri R, manual S
**23/0645**	*K. pneumoniae*	ME	amikacin	Colibri R, manual S	Colibri S, manual S
**23/1560**	*E. coli*	ME	nitrofurantoin	Colibri R, manual S	Colibri S, manual S
**Q09/035**	*P. aeruginosa*	ME	meropenem	Colibri R, manual S	Colibri S, manual S
**REF-708**	*K. pneumoniae*	ME	cefepime	Colibri R, manual S	Colibri R, manual R
**REF-716**	*K. oxytoca*	ME	piperacillin/tazobactam	Colibri R, manual S	Colibri R, manual S
**REF-717**	*K. pneumoniae*	ME	amikacin	Colibri R, manual S	Colibri R, manual R
**Q09/028**	*K. pneumoniae*	ME	cefuroxime	Colibri R, manual S	Colibri S, manual S
**Q18/011**	*K. pneumoniae*	ME	cefuroxime	Colibri R, manual S	Colibri S, manual S
**Q22/033**	*K. oxytoca*	ME	amikacin	Colibri R, manual S	Colibri R, manual R
**Q23/030**	*E. coli*	ME	amikacin	Colibri R, manual S	Colibri R, manual R

## Data Availability

The original contributions presented in this study are included in the article/Supplementary Material. Further inquiries can be directed to the corresponding authors.

## Supplementary Materials

The following supporting information can be downloaded at: https://www.mdpi.com/article/10.3390/antibiotics14121178/s1, Supplementary materials S1.
